# Supplementary material for: Preliminary Study on Light-Activated Antimicrobial Agents as Photocatalytic Method for Protection of Surfaces with Increased Risk of Infections
Source: Materials (Basel). 2021 Sep 14;14(18):5307. doi: 10.3390/ma14185307 (PMC8470258; doi:10.3390/ma14185307)
Supplement: Supplementary file 1 [file materials-14-05307-s001.zip › materials-1296796-supplementary.pdf]

materials

Supplementary Materials

Preliminary Study on Light-Activated Antimicrobial Agents as Photocatalytic Method for Protection of Surfaces with Increased Risk of Infections

Razvan Bucuresteanu [1,2], Lia-Mara Ditu [1,2,*], Monica Ionita [3], Ioan Calinescu [3], Valentin Raditoiu [4], Bogdan Cojocaru [5], Ludmila Otilia Cinteza [6], Carmen Curutiu [1,2], Alina Maria Holban [1,2], Marius Enachescu [7,8], Laura-Bianca Enache [7], Gabriel Mustatea [9], Viorel Chihaia [10], Adela Nicolaev [11], Elena-Larisa Borcan [11,12] and Grigore Mihaescu [1,2]

[1] Department of Microbiology, Faculty of Biology, University of Bucharest, Intr. Portocalelor no 1-3, 060101 Bucharest, Romania; razvan.bucuresteanu@drd.unibuc.ro (R.B.); carmen.curutiu@bio.unibuc.ro (C.C.); alina.m.holban@bio.unibuc.ro (A.M.H.); grigore.mihaescu@bio.unibuc.ro (G.M.)
[2] Faculty of Biology, Research Institute, University of Bucharest, Soseaua Paduri 90-92, 50663 Bucharest, Romania
[3] Faculty of Applied Chemistry and Materials Science, University Politehnica of Bucharest, Splaiul Independenței no 313, 060042 Bucharest, Romania; ionita_monica@yahoo.com (M.I.); ioan.calinescu@upb.ro (I.C.)
[4] Laboratory of Functional Dyes and Related Materials, National Institute for Research & Development in Chemistry and Petrochemistry—ICECHIM, 202 Splaiul Independentei, 6th District, 060021 Bucharest, Romania; vraditoiu@icechim.ro
[5] Department of Organic Chemistry, Biochemistry & Catalysis, Faculty of Chemistry, University of Bucharest, Bdul Regina Elisabeta 4-12, 030016 Bucharest, Romania; bogdan.cojocaru@chimie.unibuc.ro
[6] Department of Physical Chemistry, Faculty of Chemistry, University of Bucharest, Bdul Regina Elisabeta 4-12, 030016 Bucharest, Romania; ocinteza@gw-chimie.math.unibuc.ro
[7] Center for Surface Science and Nanotechnology, University POLITEHNICA of Bucharest, 313 Splaiul Independentei, 060042 Bucharest, Romania; marius.enachescu@cssnt-upb.ro (M.E.); laura.bianca@cssnt-upb.ro (L.-B.E.)
[8] Academy of Romanian Scientists, 54 Spaiul Independentei, 050094 Bucharest, Romania
[9] National R&D Institute for Food Bioresources—IBA Bucharest, 5 Ancuţa Băneasa Street, 020323 Bucharest, Romania; gabi.mustatea@bioresurse.ro
[10] Institute of Physical Chemistry "Ilie Murgulescu", Romanian Academy, Splaiul Independentei 202, 060021 Bucharest, Romania; vchihaia@icf.ro
[11] Department of Surfaces and Interfaces, National Institute of Materials Physics, Atomistilor 405A, 077125 Magurele, Romania; adela.nicolaev@infim.ro (A.N.); elena-larisa.borcan@drd.unibuc.ro (E.-L.B.)
[12] Faculty of Physics, University of Bucharest, Atomistilor 405, 077125 Magurele, Romania
* Correspondence: lia-mara.ditu@bio.unibuc.ro; Tel.: +40-04-0745-67-38-22

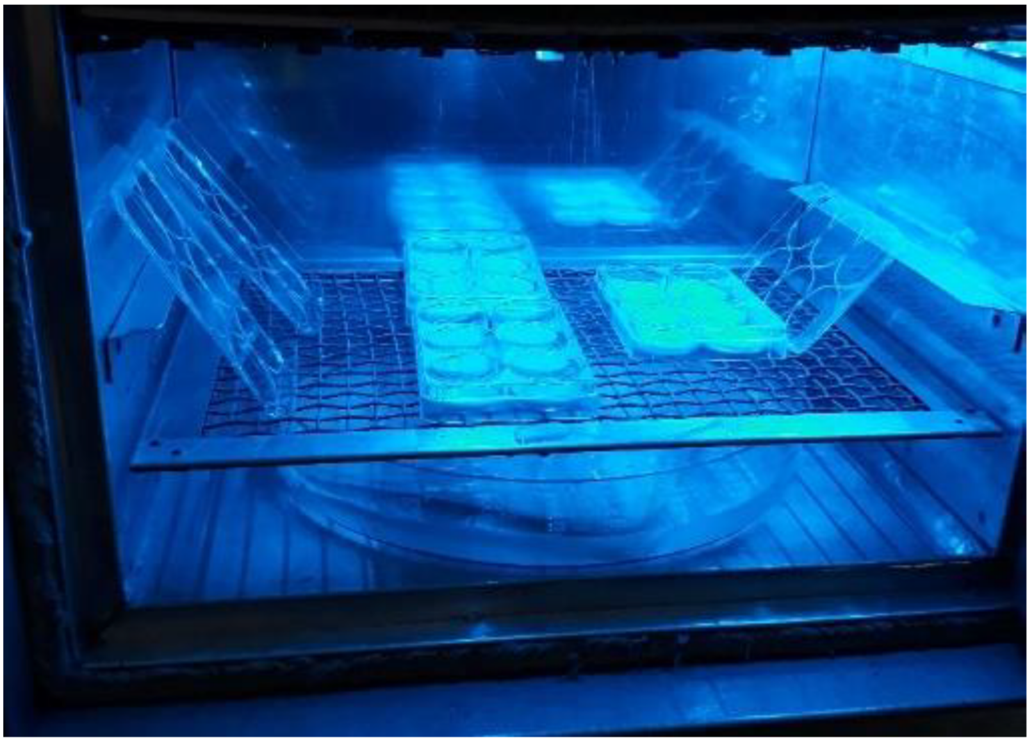

Figure S1. Samples exposure to blue light (470 nm) generated by a commercial light led source with 470 nm wavelength emission 11 spectrum.

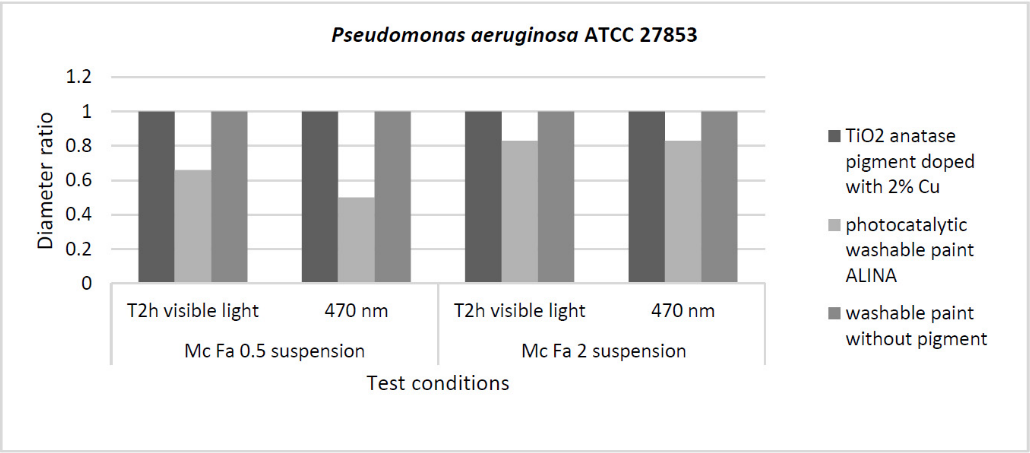

Figure S2. Graphic representation of inhibition zone diameters expressed as diameter ratio, obtained for *P. aeruginosa* ATCC 27853 15 after incubation in two different conditions: visible light and blue light (470 nm).

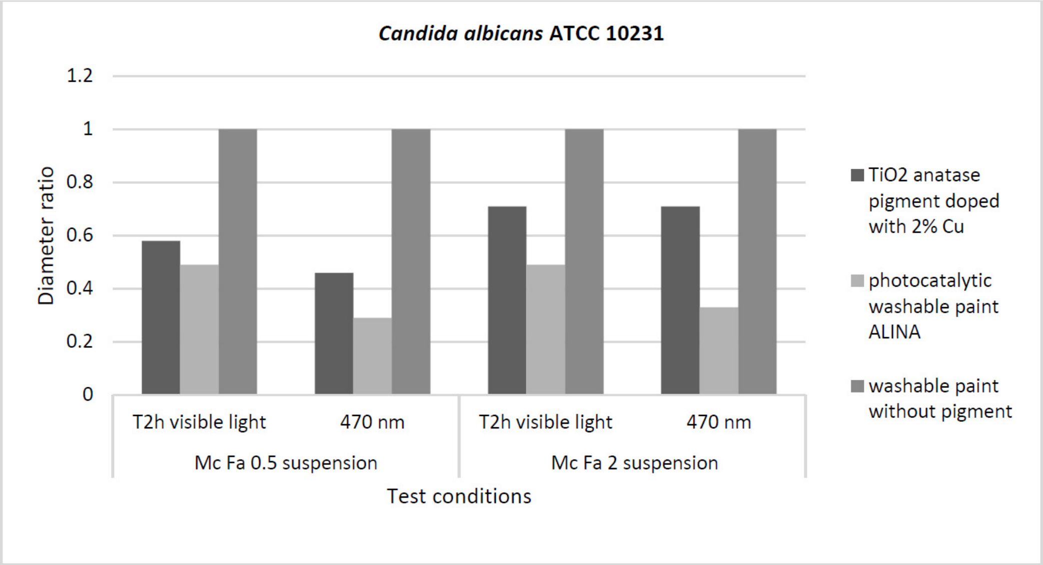

Figure S3. Graphic representation of inhibition zone diameters expressed as diameter ratio, obtained for C. albicans ATCC 10231 18 after incubation in two different conditions: visible light and blue light (470 nm).
